# A 10-year-old boy with dyspnea and hypoxia: abernathy malformation masquerading as pulmonary arteriovenous fistula

**DOI:** 10.1186/s12887-019-1422-x

**Published:** 2019-02-11

**Authors:** Lijian Xie, Yun Li, Xunwei Jiang, Jian Zhao, Tingting Xiao

**Affiliations:** Department of Cardiology, Shanghai Children’s Hospital, Shanghai Jiaotong University, No. 355 Luding Road, Shanghai, 200062 China

**Keywords:** Abernethy malformation, Hepatopulmonary syndrome, Pulmonary arteriovenous fistula

## Abstract

**Background:**

Abernethy malformation is an extremely rare congenital malformation characterised by an extrahepatic portosystemic shunt. Children with Abernathy malformation can develop hepatopulmonary syndrome (HPS) with pulmonary arteriovenous fistulas (PAVF) or pulmonary hypertension. PAVF manifests as central cyanosis with effort intolerance. We report a case of PAVF in a Ten-year-old Boy. Persistent symptoms identified Abernathy malformation as the cause of progressive symptoms and current understanding of this rare malformation is reviewed.

**Case presentation:**

A case of 10-year-old boy with Abernethy malformation complicated with HPS initially managed as PAVF was presented. Selective lung angiography showed a typical diffuse reticular pattern on right lower lung, which suggested PAVF. However, cyanosis was not improved post transcatheter coil embolization. Then, liver disease was considered although the patient had normal aspartate aminotransferase and alanine aminotransferase. The significantly elevated serum ammonia was attracted our attention. Abdominal computed tomography also exhibited enlarged main portal vein (MPV), cirsoid spleen vein, and superior mesenteric vein (SMV). Angiography with direct opacification of the SMV with a catheter coming from the inferior vena cava (IVC) and going to the SMV via the shunt vessel (SHUNT) between the MPV and IVC. Occlusion the IVC with an inflated balloon, injection of contrast medium via a catheter placed in the SMV, MPV was showed and absence of intrahepatic branches. Abernethy malformation IB type is finally confirmed.

**Conclusions:**

Abernethy malformation is an unusual cause for development of PAVF and cyanosis in children. Clinicians must be suspicious of Abernethy malformation complicated with HPS. If patients have abnormal serum ammonia and enlarged MPV in abdominal CT, cathether angiography should be done to rule out Abernethy malformation.

## Background

Abernethy malformation is an extremely rare congenital malformation characterised by an extrahepatic portosystemic shunt. It was first reported by John Abernethy and it is named Abernethy malformation [1]. Until now, more than 300 cases have been reported with a literature review, and most patients were female and less than 18 years old [2, 3]. Abernethy malformation can be classified into two types. Type I is defined by an absence of intrahepatic portal veins, and lack of liver perfusion with portal blood. Type I anomaly may be further divided into subtype A and B, defined as superior mesenteric and splenic veins draining separately into the inferior vena cava (IVC) in type IA or draining from a common trunk in type IB [4]. Type II anomaly consists of a hypoplastic portal vein supplying the liver through side-to-side anastomosis, and a larger branch that drains directly into the IVC, which means hypoplastic intrahepatic portal vein with some degree of portal flow into the liver [5].

The clinical manifestations of Abernethy malformation are highly variable and can be divided into 3 types: ①asymptomatic, ②symptoms due to the abnormal liver development such as hepatic encephalopathy or multiple liver nodules/tumors, and ③shunt related symptoms such as pulmonary hypertension or hepatopulmonary syndrome (HPS) [4, 6–8]. HPS is characterized by the presence of dyspnea and hypoxia in patients with liver diseases. Pulmonary arteriovenous fistula (PAVF) is a rare complication of Abernethy malformation, which is occurred secondary to liver failure or bypassing of the liver by portosystemic shunt [9]. PAVF is clinically and radiologically divided into simple and complex type and commonly associated with hereditary hemorrhagic telangiectasia (HHT) [10]. PAVF is not easily diagnosed routinely, due to its rarity and its unspecific findings on routine examinations [11]. Chest computed tomography (CT) and selective lung angiography are helpful to diagnose PAVF. If the patient’s clinical presentation is hypoxia caused by PAVF, its primary disease (Liver disease or Abernethy malformation) is easily to be likely to be overlooked in the initial evaluation. .

Here, a case of 10-year-old boy with Abernethy malformation complicated with HPS who is initially managed as PAVF.

## Case presentation

**Fig. 1:**
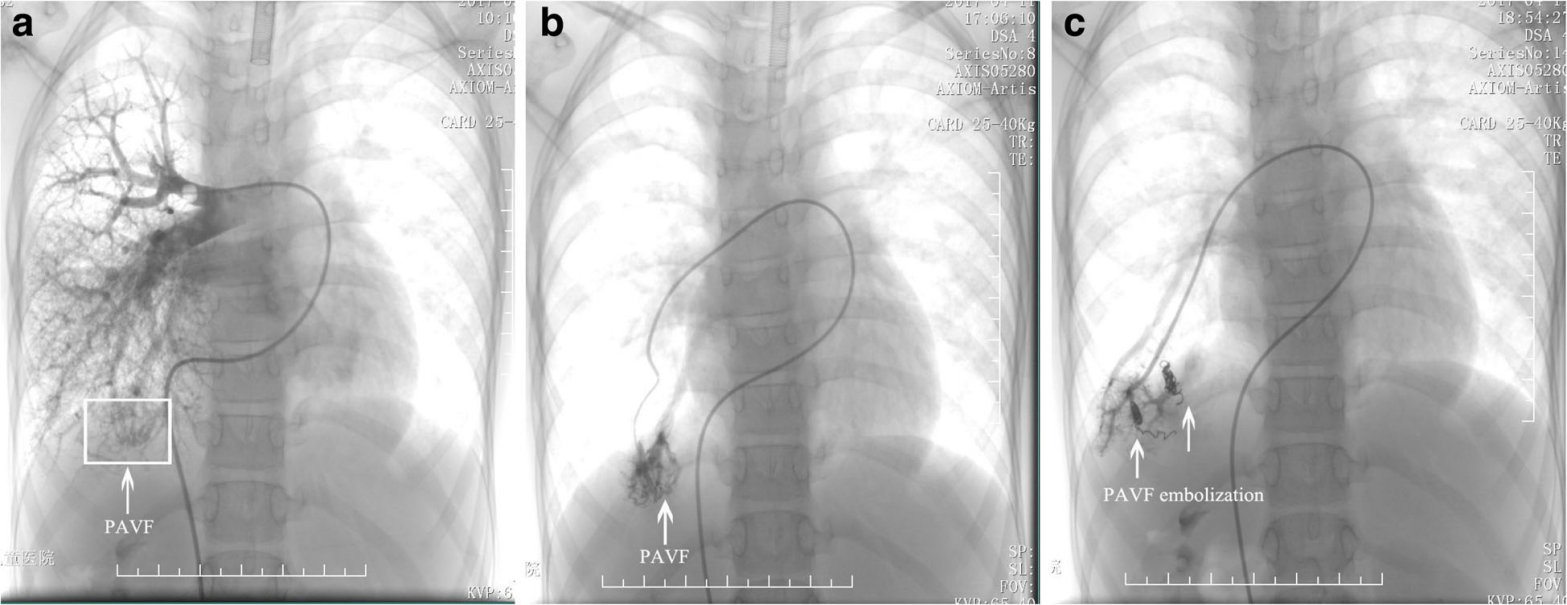
**a** A selective right pulmonary arteriogram showed diffuse reticular pattern on right lower lung, which suggested pulmonary arteriovenous fistula (PAVF). **b** Right lower pulmonary arteriogram with micro catheter showed diffuse and distorted PAVF. **c** After the coil embolization, pulmonary arteriovenous shunt was significantly reduced

A 10-year-old Chinese boy, with a height of 152 cm and weighing 35 kg, was presented with 4-year history of cyanosis and dyspnea on exertion. Physical examination on admission revealed a central cyanosis and digital clubbing with a resting pulse oximetry (SpO_2_) of 75% on room air. Chest and cardiac examination results were unremarkable. His abdominal examination showed situs solitus and no hepatomegaly. He had mild mental retardation, however, no evidence of encephalopathy. His laboratory test showed an elevated hemoglobin level of 16.5 g/L, a normal liver enzyme enzyme profile with aspartate aminotransferase 16 U/L, alanine aminotransferase 20 U/L. Direct bilirubin was 4 μmol/L (normal range 0 to 6.8 μmol/L) and albumin was 40.8 g/L (normal range 38 to 54 g/L). Chest X-ray, electrocardiogram and echocardiogram results were unremarkable. Chest CT showed diffuse pulmonary hypervascularization. Hence, diffuse PAVF was suspected. A right cardiac catheterization was performed, which showed a normal pulmonary artery pressure. Selective lung angiography showed immediate opacification of the left atrium, and typical diffuse reticular vessel pattern on right lower lung, which suggested PAVF (Fig. 1). Transcatheter coil embolization for PAVF of 7 micro coils was performed, however, pulmonary arteriovenous shunt was still existing post occlusion (Fig. 1). And, symptoms of cyanosis and dyspnea were not improved.

**Fig. 2:**
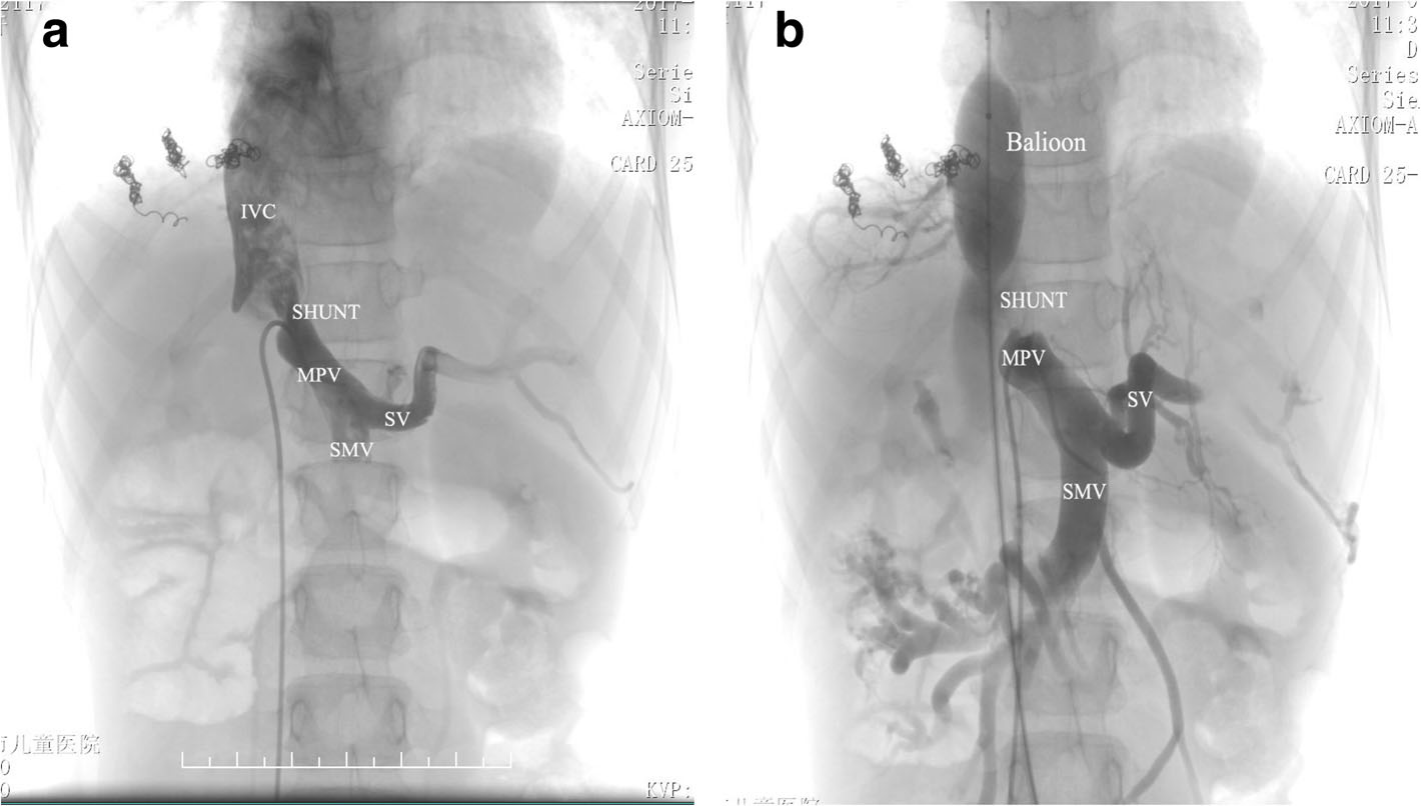
An angiogram showed side to side communication between the main portal vein (MPV) and the inferior vena cava (IVC) in our case. **a** Angiography with direct opacification of the superior mesenteric vein (SMV) with a catheter coming from the IVC and going to the SMV via the shunt vessel (SHUNT) between the MPV and IVC. SMV and splenic vein (SV) are draining from a common trunk. **b** Occlusion the IVC with a inflated balloon, injection of contrast medium via a catheter placed in the SMV, MPV was showed and no intrahepatic branches were showed. So, our case is confirmed as Abernethy malformation IB type

So, we began to suspect our original diagnosis of PAVF and liver disease was considered. We found the serum ammonia was elevated to 82 μmol/L (normal range from 16 to 60 μmol/L). The elevated serum ammonia was attracted our attention. Furthermore, abdominal contrast enhanced CT showed the main portal vein (MPV) was enlarged, spleen vein (SV) and superior mesenteric vein (SMV) and its branches were circuity expansion. Congenital extrahepatic portosystemic shunt was considered. A selective cathether angiography was subsequently performed, and the results confirmed it was Abernethy malformation IB type. Angiography with direct opacification of the SMV with a catheter coming from the IVC and going to the SMV via the shunt vessel (SHUNT) between the MPV and IVC (Fig. 2). Occlusion the IVC with an inflated balloon, injection of contrast medium via a catheter placed in the SMV, MPV was showed and no intrahepatic branches could be opacified (Fig. 2). Finally, the investigatory features were consistent with the diagnosis of type IB Abernethy malformation, and the child is now scheduled for Liver transplantation.

## Discussion and conclusions

Although overall a rare malformation, Abernethy malformation is being diagnosed more frequently with the advances in imaging techniques. Contrast CT scan and Three-dimensional (3D) vessel reconstruction could confirm Abernethy malformation diagnosis in children and adults [12–14]. The 3D reconstruction of blood vessels is very useful for demonstrating extrahepatic portocaval shunts in patients who are suspected Abernethy malformation [15]. Moreover, an experienced sonographer could detect side-to-side communication between the MPV and IVC in subcostal window [16, 17]. Anyway, selective cathether angiography in the MPV is a golden diagnostic standard.

Here we reported a 10-year-old male Abernethy malformation, which exhibited cyanosis and dyspnea. Firstly, selective lung angiography showed the appearance of dilated distal pulmonary arteries and a classic reticular pattern suggestive of PAVF. However, SpO_2_ and cyanosis were not improved post transcatheter coil embolization. We reviewed the boy’s clinical, biochemical and radiological data again. The elevated serum ammonia caused our attention. Abdominal CT also showed enlarged MPV, cirsoid SV and SMV. Finally, Abernethy malformation IB type is confirmed by cathether angiography. PAVF is secondary to Abernethy malformation complicated with HPS.

HPS is a triad of liver diseases, arterial hypoxemia and pulmonary vascular dilatation. Although HPS typically develops in the presence of cirrhosis or portal hypertension, it may also occur in the absence of parenchymal liver disease in association with portosystemic shunting. To our knowledge, Abernethy malformation with HPS was first reported in a 3-year-old girl with polysplenia syndrome in 1993 [18]. Until now, HPS has been reported in at least 20 children with Abernethy malformation [19]. Three hypothesis accounting for the etiology have been proposed, that is, elevated endothelin-1 circulating in the whole body which up-regulates nitric oxide (NO) production in the lungs by continuously stimulating the NO synthase, hepatic products necessary for pulmonary vasomotor control are decreased by liver dysfunction or hepatic venous flow reduction, ③translocation of gut bacteria activating alveolar macrophages results in increase in inducible NO synthase [13]. In a word, HPS is believed to be attributed to the exposure of the pulmonary vascular bed to vasoactive mediators, which derived from the intestinal tract entering the systemic circulation without being metabolized in the liver [3]. So, PAVF is occurred secondary to liver failure or bypassing of the liver by portosystemic shunt [5].

Treatment of congenital malformations of the portal system depends on the type of Abernethy malformation, the presenting symptoms, complications and comorbidity. Treatment may vary from surgical correction or transcatheter occlusion of the shunt to even liver transplantation [19, 20]. For pediatric patients with extrahepatic or intrahepatic portosystemic shunt, the appropriate time of therapy has not been confirmed [21]. It is proposed that even in the absence of overt symptoms, early intervention prevents pulmonary complications and neurodevelopmental delay could be avoided [22]. The clear clinical intervention indications are encephalopathy, HPS, porto-pulmonary hypertension, liver nodules regenerating, and increasing shunt size if the patient is fit to tolerate any invasive procedure [21]. The choice of transcatheter or surgical approach depends on the classification of the malformation as type I or II. The selection of percutaneous embolic materials is depending on shunt size, which included coils, detachable balloons, and vascular plugs [21]. A temporary balloon occlusion test is very important to evaluate the portal vein pressure during the intervention procedure. Surgical ligation is the another method in cases with large shunts with risk of materials migration during embolization or post embolization failure [21].

Liver transplantation has been performed for Abernethy malformation type I. Given the lack of an apparent intrahepatic portal venous system in type I disease, liver transplantation is a definitive therapy. Sakamoto et al. have reviewed 34 patients treated by liver transplantation for extrahepatic shunts. Good outcomes were reported, with 31 patients alive (91.2%) at a median follow-up of 18 months [23, 24]. Malignant tumors have been reported to occur in Abernethy malformation patients in the absence of liver dysfunction and cirrhosis (4% of all cases) [25]. Indications for liver transplant remain poorly defined except for hepatocellular carcinoma cases [23, 24].

Exploiting phenotypic plasticity for an 11-year-old boy with HPS secondary to type I Abernethy malformatio was firstly reported by Kuo et al. [26]. It is a new treatment for Abernethy malformation, which included 3-staged endovascular method. Stage 1, portal venous outflow tract establishment by creating a transjugular intrahepatic portosystemic shunt (TIPS), then a second covered stent was placed in his IVC to block the portacaval fistula. Stage 2, 6 weeks later, intrahepatic portal venous network was developed. The TIPS stent was reduced. Stage 3, next 6 weeks later, normal left and right portal veins and normal-sized intrahepatic portal veins were confirmed by venography The remaining TIPS was completely blocked with Amplatzer Vascular Plug and/or embolization coils [26]. The patient totally remains free of HPS stigmata 2 years later. This case provides a new therapy strategy in patient with Abernethy malformation and HPS.

In conclusion, via this case, we make a point that Abernethy malformation is an unusual cause for development of PAVF and cyanosis in children Clinicians must be suspicious of Abernethy malformation complicated with HPS as abnormally elevated serum ammonia levels and enlarged portal veins with demonstration of portosystemic fisulas on imaging are other diagnostic clues.

## Abbreviations

HPS: Hepatopulmonary syndrome
IVC: Inferior vena cava
MPV: Main portal vein
NO: Nitric oxide
PAVF: Pulmonary arteriovenous fistula
SMV: Superior mesenteric vein
SV: Spleen vein
